# Use of Medications for Opioid Use Disorder and Child Welfare Outcomes

**DOI:** 10.1001/jamahealthforum.2024.1768

**Published:** 2024-07-12

**Authors:** Alexandra Muhar, Elizabeth McNeer, Lauren D. Presley, Anna Morad, Sarah F. Loch, William D. Dupont, Stephen W. Patrick

**Affiliations:** 1 Department of Pediatrics, Vanderbilt University Medical Center, Nashville, Tennessee; 2 Vanderbilt Center for Child Health Policy, Vanderbilt University Medical Center, Nashville, Tennessee; 3 Department of Biostatistics, Vanderbilt University Medical Center, Nashville, Tennessee; 4 Department of Health Policy, Vanderbilt University Medical Center, Nashville, Tennessee; 5 Now with Department of Health Policy and Management, Rollins School of Public Health, Atlanta, Georgia

## Abstract

This cohort study investigates whether use of medications for opioid use disorder in pregnancy is associated with higher rates of infants discharge home with their mothers after birth.

## Introduction

The number of pregnant people with opioid use disorder (OUD) has increased substantially,^1^ with a concurrent rise in infants entering foster care due to parental substance use.^2^ Medications for OUD (MOUD) are recommended for use during pregnancy and associated with improved clinical outcomes for both mother and infant.^3,4^ Studies have shown that during pregnancy, MOUD may contribute to reductions in family separation, but they have been limited by small samples and potential selection bias.^5^ We investigated whether use of MOUD during pregnancy is associated with higher rates of infant discharge home with their biological mother.

## Methods

This cohort study included all opioid-exposed infants born at Vanderbilt University Medical Center who were at least 35 weeks’ gestational age without critical illness between March 1, 2018, and January 1, 2022. The Vanderbilt University Medical Center Institutional Review Board considered this study exempt from human participants review and waived informed consent. The study followed the STROBE reporting guideline.

Data were obtained using a standardized data collection tool during the birth hospitalization, electronic health records, and zip code–level data on treatment access experimentally derived from an audit study (eTable, eFigure 1 in Supplement 1). Propensity score overlap weighting adjusted for confounding variables, and a weighted logistic regression model evaluated the association of MOUD with infant discharge home with their mother and tested the interaction of MOUD and race (dichotomized as non-Hispanic Black and non-Hispanic White because of low numbers of other races and ethnicities) (eAppendix, eFigure 2 in Supplement 1). Race data were included because of documented racial disparities in the receipt of MOUD.

Data were analyzed using R, version 4.2.1 (R Foundation for Statistical Computing). A 2-sided *P* < .05 was considered significant.

## Results

Among 459 mother-infant dyads included, 362 mothers (78.9%; mean [SD] age, 30.1 [4.8] years) received MOUD during pregnancy, and 350 infants (76.3%) were discharged home with their mothers. Mothers who received MOUD were more likely than those who did not to be non-Hispanic White (335 [92.5%] vs 64 [66.0%]), covered by Medicaid (335 [92.5%] vs 77 [79.4%]), have infants born at higher gestational age (mean [SD], 38.5 [1.4] vs 37.6 [1.4] weeks), and be discharged home with their infants (300 [82.9%] vs 50 [51.5%]; all *P* < .001) (Table). In propensity score analysis, infants of mothers who received MOUD (81.5%; 95% CI, 72.3%-86.8%) during pregnancy were more likely to be discharged home with their mothers than infants whose mothers did not receive MOUD (51.8%; 95% CI, 41.5%-62.2%). There was no effect modification by race (Figure).

**Table.** Characteristics of Pregnant Patients Receiving and Not Receiving MOUDs^a^

Characteristic	No. of patients (%)		*P* value
	No MOUD^b^ (n = 97)	MOUD^b^ (n = 362)	
**Mother **			
Age, mean (SD), y	30.2 (5.5)	30.1 (4.8)	.98
Race			
Non-Hispanic Black	27 (27.8)	22 (6.1)	<.001
Non-Hispanic White	64 (66.0)	335 (92.5)	
Other^c^	(6.2)^d^	(1.4)^d^	
Insurance			
Medicaid	77 (79.4)	335 (92.5)	<.001
Private	(7.2)^d^	22 (6.1)	
Uninsured	13 (13.4)	(1.4)^d^	
HCV infection	12 (12.4)	96 (26.5)	.005
Mental health conditions			
Anxiety disorder	17 (17.5)	69 (19.1)	.84
Depressive disorder	10 (10.3)	43 (11.9)	.80
Bipolar disorder	(3.1)^d^	33 (9.1)	.08
Other	(2.1)^d^	(1.9)^d^	>.99
Maternal substance and medication use			
Smoking	35 (36.1)	205 (56.6)	<.001
Benzodiazepine	20 (20.6)	29 (8.0)	.001
Illicit substances	21 (21.6)	50 (13.8)	.08
Atypical antipsychotic	(7.2)^d^	45 (12.4)	.21
Typical antipsychotic	(2.1)^d^	14 (3.9)	.58
SSRI	(3.1)^d^	16 (4.4)	.77
Gabapentin	(4.1)^d^	16 (4.4)	>.99
Treatment provider access			
Treatment providers in zip code	82 (84.5)	251 (69.3)	.001
No providers in zip code	15 (15.5)	111 (30.7)	
None accept pregnant patients	44 (45.4)	100 (27.6)	
Pregnant patients accepted	38 (39.2)	151 (41.7)	
**Infant**			
Gestational age at birth, mean (SD), wk	37.6 (1.4)	38.5 (1.4)	<.001
Final disposition^e^			
Discharged home	72 (74.2)	336 (92.8)	<.001
Discharged home with mother^f^	50 (51.5)	300 (82.9)	
Foster care	18 (18.6)	18 (5.0)	
Adoption	(7.2)^d^	7 (1.9)	
Other	0	(0.3)^d^	

Abbreviations: HCV, hepatitis C virus; MOUD, medication for opioid use disorder; SSRI, selective serotonin reuptake inhibitor.

^a^ Data were obtained during the birth hospitalization using a standardized data collection process and were augmented with data from electronic medical records and on treatment access from a secret shopper study of pregnant people with opioid use disorder.

^b^ Medications for opioid use disorder were defined as using an MOUD on admission to the birth hospitalization.

^c^ Includes unspecified and biracial or multiracial.

^d^ Counts are suppressed because the number of individuals was less than 10.

^e^ Discharge data, including discharge home with biological mother, were coded by the treatment team during the birth hospitalization using a standardized data collection process. Infant was discharged at the end of the birth hospitalization. Discharge home can be with the biological mother and/or in kinship care.

^f^ Discharged home with biological mother, not in kinship care, foster care, adopted, transferred, or died.

**Figure.**
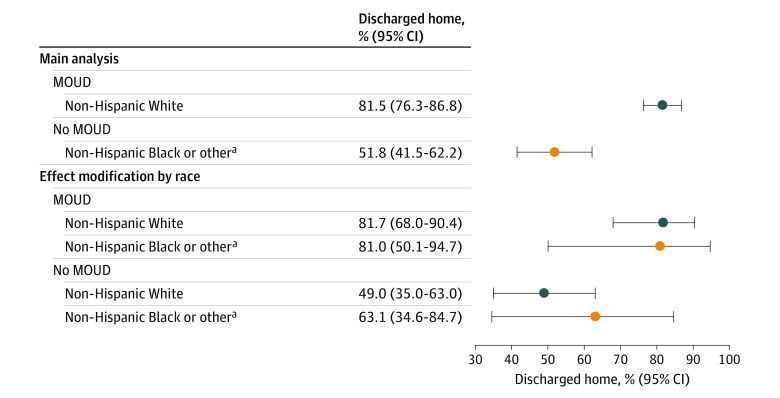
Association of Medications for Opioid Use Disorder (MOUD) During Pregnancy With Adjusted Probability of Infant Discharge Home With Their Biological Mother and Effect Modification of Race and MOUD Receipt These propensity score analyses are adjusted for confounding variables using propensity scores with overlap weighting. Cohorts (MOUD vs no MOUD) were balanced based on variables that may influence access to treatment, including access to care (buprenorphine providers in county who accept pregnant people, insurance type), smoking, antipsychotic use, selective serotonin reuptake inhibitor use, gabapentin use, hepatitis C viral infection status, mental health conditions (depression, anxiety, bipolar, other), and race. ^a^Includes unspecified and biracial or multiracial.

## Discussion

Our findings show that infants of mothers who received MOUD during pregnancy were more likely to be discharged home with their mothers, reinforcing prior work that suggested improved retention of custody for parents who receive MOUD.^5^ The decision to place a child into foster care is complex, and MOUD is only 1 factor in a comprehensive risk assessment. Although we found that Black mothers were less likely to receive MOUD than White mothers, there was no effect modification with MOUD receipt and infants being discharged home with their mother.

Gaps in research on MOUD and child welfare outcomes may create barriers to funding for support services for families with OUD. The Family First Prevention Services Act allows states to use Title IV-E federal foster care funds to support programs that prevent foster care entry.^6^ Programs must meet evidence-based criteria to qualify for the funds; however, despite current literature and guidelines for MOUD treatment in pregnancy,^3^ buprenorphine is not an approved service because of a limited association with improved child welfare outcomes.

Our study is limited by lack of generalizability, misclassification bias, and heterogeneity in the treatment group, as well as a predominately non-Hispanic White sample, which may have reduced our power to detect effect modification by race. Nonetheless, we provide additional evidence of positive pregnancy outcomes with MOUD use that extend beyond traditionally studied clinical outcomes for child welfare.

## Notes

Supplement 1.
eAppendix
**eTable.** Data Sources and Definitions **eFigure 1.** Clinical Data Collection Sheet **eFigure 2.** Love Plot Showing Covariate Balance Between the Treated and Untreated Groups With and Without Inverse Probability and Overlap Weighting

Supplement 2.
Data Sharing Statement
