# Supplementary material for: Use of Medications for Opioid Use Disorder and Child Welfare Outcomes
Source: JAMA Health Forum. 2024 Jul 12;5(7):e241768. doi: 10.1001/jamahealthforum.2024.1768 (PMC11245717; doi:10.1001/jamahealthforum.2024.1768)
Supplement: Supplement 1. — eAppendix eTable. Data Sources and Definitions eFigure 1. Clinical Data Collection Sheet eFigure 2. Love Plot Showing Covariate Balance Between the Treated and Untreated Groups With and Without Inverse Probability and Overlap Weighting [file jamahealthforum-e241768-s001.pdf]

Supplemental Online Content

Muhar A, McNeer E, Presley LD, et al. Use of medications for opioid use disorder and child welfare outcomes. *JAMA Health Forum*. 2024;5(7):e241768. doi:10.1001/jamahealthforum.2024.1768

eAppendix

eTable. Data Sources and Definitions

eFigure 1. Clinical Data Collection Sheet

eFigure 2. Love Plot Showing Covariate Balance Between the Treated and Untreated Groups With and Without Inverse Probability and Overlap Weighting

This supplemental material has been provided by the authors to give readers additional information about their work.

eAppendix

Statistical Approach
Logistic regression was used to derive propensity scores by regressing MOUD against the other variables listed in Appendix Table 1 as being included in this score. Indicator covariates were used for categorical variables. Overlap weights were derived as described in Thomas et al. *JAMA* 2020; **323**(23): 2417-2418. The PSweight package in R was used to calculate the overlap weights. The marginal effect of MOUD on the probability for home discharge was estimated by regressing home discharge against MOUD using these overlap weights.

Missing Data
If a patient was missing a value for an indicator variable (smoking, HCV infection, illicit substance use, etc.), we set the variable to 0 since we did not have evidence that the risk factor was present. For the insurance variable, we filled in missingness from the clinical data collection using data from the VUMC EHR. The clinical data collection had a Yes/No variable for TennCare/Medicaid recipient. For the 13.1% of patients who had a "No" for this variable and the 0.4% of patients who were missing a value for this variable, we used insurance data from the VUMC EHR.

Effect of Race
An assessment of the effect of race/ethnicity on home discharge was estimated by regressing home discharge against MOUD, race (dichotomized as non-Hispanic white vs non-white) and a MOUD-race interaction term using the overlap weights described above. Race was included in the calculation of the overlap weights in order to increase the exchangeability of treated and untreated mothers as recommended by Hernan et al. *JAMA* 2022; **328**(24): 2446-2447.

Limitations
Our study has limitations which merits mentioning, including generalizability, misclassification bias, heterogeneity in the treatment group, and a sample that was disproportionately non-Hispanic white.

- Generalizability: Given that our study occurred at a single center, it is possible that our results are not generalizable outside of this setting.
- Misclassification bias: Despite using a standardized data collection sheet, a standard process to collect real-time clinical data, and processes to validate data, it is possible that data could be miscoded leading to errors of omission or commission.
- Heterogeneity in the treatment group: We gathered data for MOUD at the time of delivery. The majority of patients in the MOUD group were treated with buprenorphine, however, we included both buprenorphine and methadone in the MOUD groups. Given this designation we are not able to evaluate differential effects between the two medications.
- Sample diversity: Our sample was disproportionately non-Hispanic white which is representative of diagnoses of OUD in pregnancy in Tennessee. The relatively small sample of non-white patients may limit our ability to evaluate effect modification by race.

© 2024 Muhar A et al. *JAMA Health Forum*.

eTable. Data Sources and Definitions

**Variable**	**Source**	**Definition**	**Used in Propensity Score Model?**
**Selection Criteria**			
Infant date of birth	Clinical Data Collection	Born between<br>March 1, 2018 and January 1, 2022	--
Infant critical illness	Clinical Data Collection	NICU length of stay ≤5 days	--
Gestational age	Clinical Data Collection	≥35 weeks	--
Opioid exposure	Clinical Data Collection	Clinically diagnosed as opioid exposed newborn	--
**Model Variables**			
Maternal receipt of MOUD	Clinical Data Collection	MOUD Used On Admission to Labor and Delivery (Figure 1)	Outcome
Gestational Age	Clinical Data Collection	Estimated Gestational Age at Birth (Figure 1)	No
Discharge to Biological Mother	Clinical Data Collection	Infant Discharged Home with Biological Mother and not in foster or kinship care	No
Treatment Providers in Zip Code	Secret Shopper[1]	Using Data Derived from A Randomized Secret Shopper Study<br>0 No Treatment Provider<br>1 Treatment Provider in Zip Code<br>2 Treatment Provider in Zip Code Accepting Pregnant Women	Yes
Insurance	Clinical Data Collection and VUMC EHR[2]	Mother's primary insurance at delivery<br>1 Private<br>2 Medicaid<br>3 Uninsured	Yes
Smoking	VUMC EHR	Mother's smoking status during pregnancy obtained from social history	Yes
Atypical Antipsychotic	VUMC EHR	Any atypical antipsychotic ordered inpatient or prescribed outpatient from 90 days to 2 days prior to delivery	Yes
Typical Antipsychotic	VUMC EHR	Any typical antipsychotic ordered inpatient or prescribed outpatient from 90 days to 2 days prior to delivery	Yes
SSRI	VUMC EHR	Any SSRI antidepressant ordered inpatient or prescribed outpatient from 90 days to 2 days prior to delivery	Yes
Benzodiazepine	VUMC EHR	Any benzodiazepine ordered inpatient or prescribed outpatient from 90 days to 2 days prior to delivery; OR<br><br>Present/positive/non-zero urine toxicology reports for benzodiazepines (Alprazolam, Alpha-OH-alprazolam, Clonazepam, 7-aminoclonazepam, Diazepam, Desalkylflurazepam, Alpha-OH-ethylflurazepam, Lorazepam, Midazolam, Nordiazepam, Oxazepam, Temazepam, Alpha-OH-triazolam) from 30 days prior to delivery up through and including birth hospitalization	Yes
Gabapentin	VUMC EHR	Any gabapentin ordered inpatient or prescribed outpatient from 90 days to 2 days prior to delivery	Yes
HCV Infection	VUMC EHR	Lab testing for maternal HCV in the prenatal period<br>0 Negative<br>1 Positive; OR	Yes

© 2024 Muhar A et al. *JAMA Health Forum.*

		Diagnostic code(s)[3] for HCV (070.41, 070.44,070.51, 070.54, 070.70, 070.71, V02.62, Z22.50, Z22.52, Z22.59, Z86.19, B17.10, B17.8, B18.2, B19.20, B19.21) in the prenatal period<br>0 No<br>1 Yes	
Illicit Substance	VUMC EHR	Present/positive/non-zero urine toxicology reports for<br>cocaine (cocaine, Benzoylecgonine (BE), Meta-OH-BE), amphetamines (Amphetamine, Methamphetamine, MDMA, MDA, MDEA), barbiturates (Butalbital, Phenobarbital, Secobarbital), cannabinoids (THC-COOH, Marijuana), opiates (fentanyl and heroin) from 30 days prior to delivery up through and including birth hospitalization<br>0 Negative<br>1 Positive	Yes
Depressive Disorders	VUMC EHR	Diagnostic code(s)[3] for depressive disorders (296.2, 296.21, 296.22, 298.23, 296.24, 296.25, 296.26, 296.3, 296.31, 296.32, 296.33, 296.34, 296.35, 300.4, 311, F32.0, F32,1, F32.2, F32.3, F32.4, F32.89, F32.9, F32.A, F33.0, F33.1, F33.2, F33.3, F33.40, F33.41, F33.8, F33.9, F34.1) within the prenatal period and 1-year postpartum period<br>0 No<br>1 Yes	Yes
Anxiety Disorders	VUMC EHR	Diagnostic code(s)[3] for anxiety disorders (293.xx, 300, 300.01, 300.02, 300.09, 300.1, 300.2, 300.21, 300.22, 300.23, 300.29, 300.3, 300.5, 300.89, 300.9, 308, 308.1, 308.2, 308.3, 308.4, 308.9, 309.81, F40.00, F40.01, F40.02, F40.10, F40.11, F40.210, F40.218, F40.220, F40.228, F40.230, F40.231, F40.232, F40.233, F40.240, F40.241, F40.242, F40.243, F40.248, F40.290, F40.291, F40.298, F40.8, F40.9, F41.0, F41.1, F41.3, F41.8, F41.9, F42, F42.2, F42.3, F42.4, F42.8, F42.9, F43.1, F43.10, F43.11, F43.12, F44.9, F45.8, F48.xx, F48.8, F48.9, F93.8, F99, R45.xx, R45.2, R45.5, R45.6, R45.7) within the prenatal period and 1-year postpartum period<br>0 No<br>1 Yes	Yes
Bipolar Disorder	VUMC EHR	Diagnostic code(s)[3] for bipolar disorder (F30.10, F30.11, F30.12, F30.13, F30.2, F30.3, F30.4, F30.8, F30.9, F31.0, F31.10, F31.11, F31.12, F31.13, F31.2, F31.30, F31.31, F31.32, F31.4, F31.5, F31.60, F31.61, F31.62, F31.63, F31.64, F31.70, F31.71, F31.72, F31.73, F31.74, F31.75, F31.76, F31.77, F31.78, F31.81, F31.89, F31.9, F33.8, F34.81, F34.89, F34.9, F39)	Yes

© 2024 Muhar A et al. *JAMA Health Forum.*

		within the prenatal period and 1-year postpartum period<br>0 No<br>1 Yes	
Other Disorder	VUMC EHR	Diagnostic code(s)[3] for schizophrenia (295, 295.01, 295,02, 295.03, 295.04, 295.05, 295.1, 295.11, 295.12, 295.13, 295.14, 295.15, 295.2, 295.21, 295.22, 295.23, 295.24, 295.25, 295.3, 295.31, 295.32, 295.33, 295.34, 295.35, 295.4, 295.41, 295.42, 295.43, 295.44, 295.45, 295.5, 295.51, 295.52, 295.53, 295.54, 295.55, 295.6, 295.61, 295.61, 295.62, 295.63, 295.64, 295.65, 295.7, 295.71, 295.72, 295.73, 295.74, 295.75, 295.8, 295.81, 295.82, 295.83, 295.84, 295.85, 295.9, 295.91, 295.92, 295.93, 295.94, 295.95, F20.0, F20.1, F20.2, F20.3, F20.5, F20.81, F20.89, F20.9, F25.0, F25.1, F25.8, F25.9), personality disorders (301.0, 301.10, 301.11, 301.12, 301.13, 301.20, 301.21, 301.22, 301.3, 301.4, 301.50, 301.51, 301.59, 301.6, 301.7, 301.81, 301.82, 301.83, 301.84, 301.89, 301.9, F21, F34.0, F34.1, F60.0, F60.1, F60.2, F60.3, F60.4, F60.5, F60.6, F60.7, F60.81, F60.89, F60.9, F68.10, F68.11, F68.12, F68.13, F69), other psychotic disorders (293.81, 293.82, 297.0, 297.1, 297.2, 297.3, 297.8, 297.9, 298.0, 298.1, 298.2, 298.3, 298.4, 298.8, 298.9, F06.0, F06.2, F21, F22, F23, F24, F28, F29, F32.3, F33.3, F44.89), and other mental health disorders (F53.1, O90.6, O99.34, 48.4, F05, F30, F34.1, G47.9, R53.81, R53.83, R45) within the prenatal period and 1-year postpartum period<br>0 No<br>1 Yes	Yes
Maternal Race	Clinical Data Collection	White<br>Black or African American<br>Other	Yes, combined with Ethnicity
Maternal Ethnicity	Clinical Data Collection	Hispanic or Latino:<br>Yes<br>No<br>Unknown	Yes, combined with Race

[1]Patrick SW, Richards MR, Dupont WD, McNeer E, Buntin MB, Martin PR, Davis MM, Davis CS, Hartmann KE, Leech AA, Lovell KS, Stein BD, Cooper WO. Association of Pregnancy and Insurance Status With Treatment Access for Opioid Use Disorder. *JAMA Open*. 2020 Aug 3;3(8):e2013456.

[2] Data from the VUMC EHR were abstracted using a fully identified database maintained for research that is comprised of elements from the clinical record. The study utilized structured data including patient-level diagnostic and procedure codes, prescription data, and laboratory testing and associated dates of care and/or testing for the maternal variables listed above. An existing linkage between maternal and infant records plus the availability of patient-level identifiers allowed the research team to link VUMC EHR data to additional clinically collected data on the infant and mother described below (Appendix Figure 1).

Danciu I, Cowan JD, Basford M, Wang X, Saip A, Osgood S, Shirey-Rice J, Kirby J, Harris PA. Secondary use of clinical data: The Vanderbilt approach. Journal of Biomedical Informatics. 2014;52: 28-35, ISSN 1532-0464,

https://doi.org/10.1016/j.jbi.2014.02.003.

[3]1 inpatient or 2 outpatient ICD-9/ICD-10 codes

© 2024 Muhar A et al. *JAMA Health Forum.*

eFigure 1. Clinical Data Collection Sheet

The data collection sheet was used by a clinical program that cared for infants born with opioid-exposure at Vanderbilt University Medical Center between 2017 and 2022. All data were collected prospectively as part of a larger quality improvement effort through an interdisciplinary model of care encompassing pediatrics, obstetrics, child life, and lactation called Team Hope.[1] Infants in the program were cared for in the newborn nursery or as a pediatric inpatient and were identified by clinical providers as opioid-exposed by staff by medical history, self-report by mother, and augmented by toxicology testing for opioids. Clinical identification of opioid-exposure in this program was used as selection criteria for inclusion in this study. Additional infant and birthing parent data, including MOUD treatment exposure and discharge outcomes for this study, were collected through maternal history and/or infant admission or manually abstracted from the VUMC EHR using the data collection sheet below. Data collected using this sheet were entered in a REDCap database.

[1]Crook TW, Munn E, Scott TA, Morad A, Wyatt J, Johnson DP, White M, Patrick SW. Improving the Discharge Process for Opioid Exposed Neonates. *Hospital Pediatrics*. 2019 Aug;9(8):643-648.

© 2024 Muhar A et al. *JAMA Health Forum.*

OPIOID EXPOSED NEWBORNS (OEN)

REDCap Record ID __________

OPIOID EXPOSED NEWBORNS *(captured on all OEN born/admitted)*

NEWBORN'S BASIC INFORMATION

Newborn's MRN ___________

Name (eg, "BB Smith" or "BG Smith") ___________

Date of birth *(MM/DD/YYYY)* ___________

Gestational Age *(completed weeks)* ___________

Mother's zip code of residence (on newborn record) ___________

TRANSFERS

Transfer in from outside facility? ○ No ○ Yes

If Yes –

→ Date of admission (MM/DD/YYYY) ___________

→ Day of life (DOL) at admission *(calculated field – not editable)*

→ Reason for transfer related to management of NAS/NOWS? ○ No ○ Yes

If No –

→ Mother's MRN ___________

DISPARITIES

Mother's race (*NOTE: "Other" includes unspecified and bi-/multi-racial)*
○ White ○ Black or AA ○ Other

Mother's ethnicity – Hispanic or Latino? ○ No ○ Yes ○ Unknown

Medicaid/TennCare recipient? ○ No ○ Yes ○ Unknown

OPIOID EXPOSURES

Clinically diagnosed as "Opioid Exposed Newborn" (OEN)? ○ No ○ Yes

If No –

! STOP DATA COLLECTION HERE !
! We do not wish to capture any data on newborns that are not opioid exposed. !
! Do not save this record. If you already have, please contact Theresa Scott so she can delete it. !

If Yes –

→ What were the maternal-fetal opioid exposures? *(check all that apply)*

- ☐ Buprenorphine *(includes Subutex and Suboxone)*
- ☐ Heroin
- ☐ Methadone
- ☐ Fentanyl ***(ADDED AS OF JANUARY 1, 2021)***
- ☐ Other Opiates *(Codeine, Hydrocodone, Hydromorphone (Dilaudid), Morphine, Oxycodone, Oxymorphone, Propoxyphene)*

→ Is mother on MAT? ○ No ○ Yes

→ Is mother a DDC/VMARP patient? ○ No ○ Yes

Notes regarding maternal history of opioid exposure during this pregnancy:

© 2024 Muhar A et al. *JAMA Health Forum.*

NON-OPIOID EXPOSURES ***(TO BE CAPTURED ON ALL OEN AS OF JANUARY 1, 2021)***

Other non-opioid maternal-fetal exposures *(check all that apply)*
☐ *NONE* ☐ Benzodiazepines ☐ Any antidepressants ☐ Nicotine ☐ Other

If Other –

→ Additional non-opioid maternal-fetal exposure *(check all that apply)*
☐ Amphetamine ☐ Cannabinoids or THC ☐ Cocaine ☐ Gabapentin ☐ Methamphetamine
☐ Phencyclidine (PCP) ☐ Other – *specify* ______________________________

Notes regarding other non-opioid maternal-fetal exposures:

OPIOID WITHDRAWAL (NAS / NOWS) ***(TO BE CAPTURED ON ALL OEN AS OF JANUARY 1, 2021)***

Ever assessed/scored for NAS/NOWS? ○ No ○ Yes

Clinically diagnosed with NAS/NOWS *(i.e. based on signs and symptoms of NAS/NOWS; not treatment dependent)* ○ No ○ Yes

(READ ONLY) **PHARMACOLOGIC TREATMENT**
NOTE: This data is generated by an Epic report and imported after the newborn has been discharged – the values are not editable.
NOTE: This data is available on all OEN born on/after Jan 1, 2018 who received morphine orally.

Did this newborn ever receive morphine orally (at least one scheduled or "unscheduled" PRN/rescue dose)? No / Yes

If Yes –

→ *Sum of scheduled morphine administrations*

→ *Count of PRN or once morphine administrations*

→ *First recorded dosing weight (kg)*

→ *First recorded non-dosing weight (kg; for those with no recorded 'dosing weight')*

→ *Clonidine was administered (Y/N)*

→ *IV morphine was administered (Y/N)*

LOCATIONS OF CARE ***(TO BE CAPTURED ON ALL OEN AS OF JANUARY 1, 2021)***

Was the newborn ever admitted to the NICU? ○ No ○ Yes

If Yes –

→ Date of admission of admission to NICU (MM/DD/YYYY) __________

→ Date of discharge from NICU (MM/DD/YYYY) __________

→ Length of stay (LOS) in NICU (days) *(calculated field – not editable)*

Was the newborn ever admitted to Pediatric Medicine & Acute Care (PMAC)? ○ No ○ Yes

If Yes –

→ Date of admission of admission to PMAC (MM/DD/YYYY) __________

→ Date of discharge from PMAC (MM/DD/YYYY) __________

→ Length of stay (LOS) in PMAC (days) *(calculated field – not editable)*

Location(s) of care *1 / 2 / 3 (calculated field – not editable; 1 =* Nursery ONLY; 2 = NICU *ONLY; 3 =* Nursery & NICU mix or NAS/NOWS specific units or Peds floors)

© 2024 Muhar A et al. *JAMA Health Forum.*

TEAM HOPE ELIGIBILITY

INCLUSION CRITERIA:
(1) EGA at least 35 & 0 weeks;
(2) Born at VUMC or transferred in from an outside facility before DOL 5;
(3) NICU LOS ≤ 5 days; and
(4) Opioid exposed.

Does this newborn meet the inclusion criteria? *0 / 1 (calculated field – not editable; 0 = No; 1 = Yes)*

If No → *! STOP DATA COLLECTION !*

If Yes → *! CONTINUE DATA COLLECTION !*

TEAM HOPE NEWBORNS *(captured on those OEN that meet Team Hope criteria)*

NON-PHARMACOLOGIC CARE

(TO BE CAPTURED AS OF JANUARY 1, 2021)

Which of the following non-pharmacologic care did this newborn receive? *(check all that apply)*

☐ Physical Therapy / Occupational Therapy (PT / OT) ☐ Lactation Support
☐ *NONE (cannot be indicated in combination with any other values)*

Did this newborn room-in? *Defined as OEN stays in a private room (on postpartum ward, NICU, or pediatric inpatient ward) with one or more caregivers for at least a portion of the newborn's admission.*

○ Yes ○ No

If Yes –

→ With who? *(check all that apply)*
☐ Biological mother ☐ Biological father
☐ Other biological caregiver(s) *(e.g. biological grandparent(s))*
☐ Foster caregiver(s) ☐ Adoptive caregiver(s)
☐ Other – *specify* ____________________________

→ Length of rooming-in?
○ Entire stay *(until newborn discharge)* ○ Partial *(only until maternal discharge)*

→ *If Partial*, notes regarding "partial" rooming-in: ***(TO BE CAPTURED AS OF JANUARY 1, 2021)***

If No –

→ Why not? ○ Rooming-in not available ○ Biological mother not eligible
○ Biological mother eligible but declined
○ Other – *specify* ____

(READ ONLY) MOTHER'S MILK & LACTATION SUPPORT

NOTE: This data is captured in a separate REDCap project and imported – the values are not editable.

Was the newborn eligible to receive their mother's milk? No / Yes

If Yes –

→ *Did the newborn ever receive their mother's milk during their hospitalization (either breastfed or fed Expressed Breast Milk (EBM))? No / Yes*

→ *Was newborn receiving their mother's milk at the time of the newborn's discharge (breastfeeding and/or (EBM))? No / Yes*

Was the mother first seen by Lactation Support at the DDC/VMARP (ie, prenatally)? No / Yes

© 2024 Muhar A et al. *JAMA Health Forum*.

DISCHARGE

Date of newborn's discharge from hospital (D/C; MM/DD/YYYY) __________

Location at D/C ○ Newborn ○ NICU ○ PMAC

(Overall) Length of stay (LOS) *(calculated field – not editable)*

Final disposition ○ Discharge to home ○ Transfer to outside facility ○ Foster care (DCS)
○ Adoption ○ Died ○ Other – *specify*____________________

If "Discharge to home", discharged to biological mother? ○ No ○ Yes

DCS report made during mother's and/or newborn's hospitalization? ○ No ○ Yes ○ Unknown

Which were "completed" by the time the newborn was discharged?

1. Follow-up appointment scheduled with Primary Care Provider (PCP)
   ○ Yes ○ No ○ Unable to determine

   → *If No* – What wasn't appointment scheduled? ____

2. Referral to home visiting services ***offered*** (e.g. CHANT, NFN, other programs)
   ○ Yes ○ No ○ Unable to determine ○ No referral due to discharge to safety placement

   → *If No* – Why wasn't referral to home visiting services offered? ___________________________________

3. Referral to early intervention services (e.g. TEIS) ○ Yes ○ No ○ Unable to determine

   → *If No* – What wasn't referral to early intervention services made? ________________________________

4. Referral to Pediatric development clinic/specialists (if diagnosed with NAS)
   ○ Yes ○ No ○ Unable to determine ○ Not indicated

   → *If No* – What wasn't referral to development clinic/specialist made? ______________________________

5. Referral for follow-up with Pediatric Gastroenterology or Infectious Disease clinic for testing of Hepatitis C Virus (HCV; if exposed) ○ Yes ○ No ○ Unable to determine ○ Not indicated

   → *If No* – What wasn't referral to follow-up made? ____

RE-ADMISSIONS

Was the newborn re-admitted within 7 days of discharge? ○ No ○ Yes

If Yes –

→ Reason for re-admission:

NOTES

Any additional notes regarding the newborn and/or mother:

© 2024 Muhar A et al. *JAMA Health Forum.*

eFigure 2. Love Plot Showing Covariate Balance Between the Treated and Untreated Groups With and Without Inverse Probability and Overlap Weighting

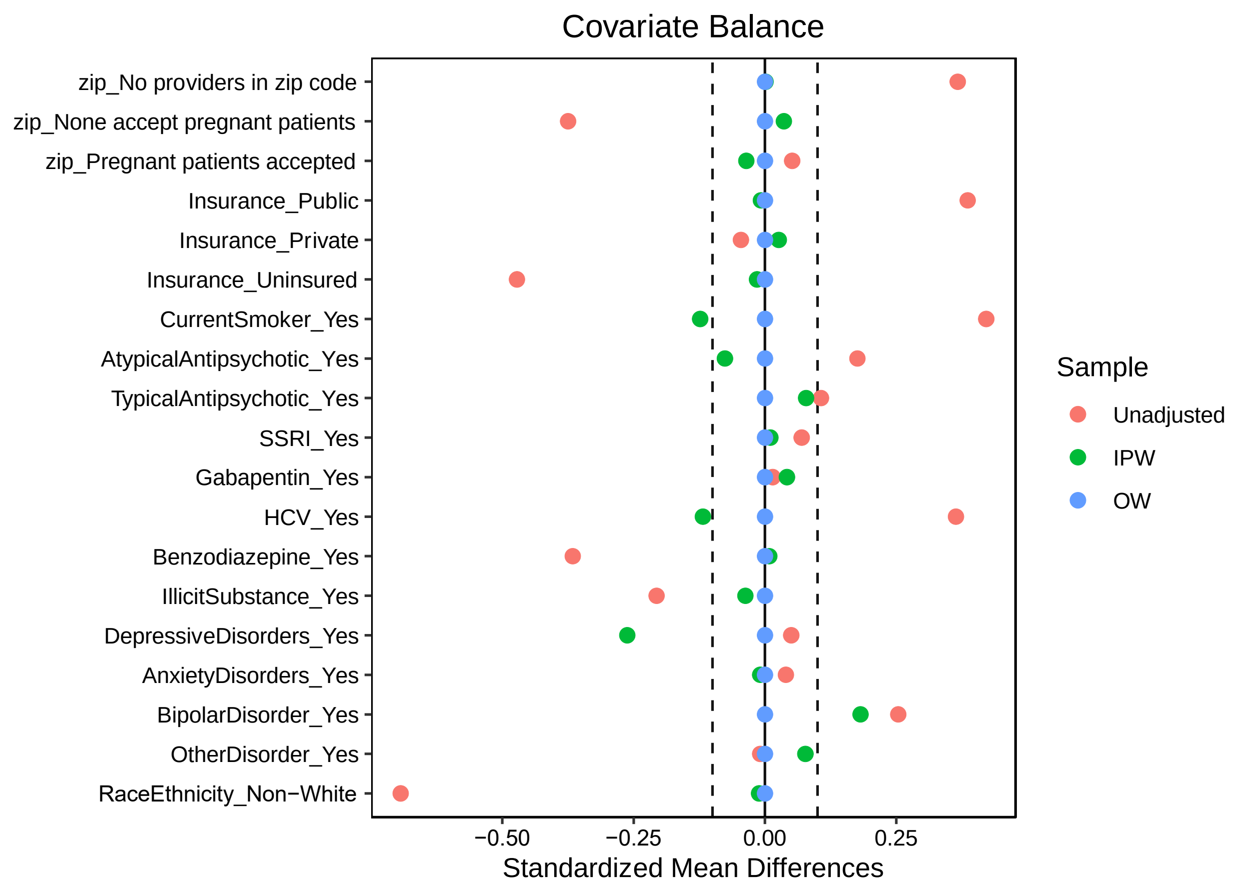

** Given superior covariate balance with overlap weighting, the primary analysis was conducted with overlap weighting rather than inverse probability weighting.

© 2024 Muhar A et al. *JAMA Health Forum.*
