# Supplementary material for: Use of Medications for Opioid Use Disorder and Child Welfare Outcomes
Source: JAMA Health Forum. 2024 Jul 12;5(7):e241768. doi: 10.1001/jamahealthforum.2024.1768 (PMC11245717; doi:10.1001/jamahealthforum.2024.1768)
Supplement: Supplement 2. — Data Sharing Statement [file jamahealthforum-e241768-s002.pdf]

Data Sharing Statement

Muhar. Use of Medications for Opioid Use Disorder and Child Welfare Outcomes. *JAMA Health Forum*. Published July 12, 2024. doi:10.1001/jamahealthforum.2024.1768

Data
Data available: Yes
Data types: Data dictionary
How to access data: In the supplemental appendix, we have provided data collection sheets and data definitions. We cannot share individual level data.
When available: With publication

Supporting Documents
Document types: Other (please specify)
Additional Information: Supplemental appendix with data definitions, data collection sheet, and details of propensity score creation with overlap weights.
How to access documents: As a supplemental appendix
When available: With publication

Additional Information
Who can access the data: These will be published as an appendix with the data, raw data are not available.
Types of analyses: As above
Mechanisms of data availability: As above, with publication as an appendix.
